# Investigation of a novel high-efficiency ion-permselective membrane module based on the electrochemically switched ion exchange scheme†

**DOI:** 10.1039/d1ra00924a

**Published:** 2021-06-16

**Authors:** Di Zhang, Pengle Zhang, Du Xiao, Xiaogang Hao

**Affiliations:** Department of Chemistry, Shanxi Medical University 56 Xinjian South Road Taiyuan 030001 China; Department of Chemical Engineering, Taiyuan University of Technology Taiyuan 030024 China xghao@tyut.edu.cn

## Abstract

Electric field-accelerated ion-permselective membrane (EISM) separation has attracted significant attention in recent years. Thus, herein, to further investigate the ion transport mechanism and optimize the separation efficiency, five types of ion-permselective membrane modules (IPMM I–V) based on the electrochemically switched ion exchange (ESIX) scheme were designed. Compared with the traditional ion separation systems, the *in situ* membrane-based ion separation system was set up with an extra pulse potential applied to the PPy/PSS/SSWM (polypyrrole/polystyrenesulfonate/stainless steel wire mesh) membrane. The continuous permselective separation of K^+^ as target ions was performed from dilute aqueous solution through the IPMM system. The pulse potential combined with the regulated cell voltage was functionalized synergistically to create an “ion-sieving effect” and effectively guide the target cations from the source cell to the receiving cell. Moreover, the formation of an equal potential volume in IPMM-V suppressed the reverse migration of the target ions and the detected ion flux across the membrane was 100 times that of the IPMM-I system. The ion transport mechanism was also analyzed in detail based on the equivalent circuit of the system, and the optimized operation parameters were obtained for the high-efficient ion separation system. These results can provide some beneficial information for the design and practical operation of novel EISM systems.

## Abstract 2

Novel ion permselective membrane modules based on the ESIX scheme.

## Graphical abstract

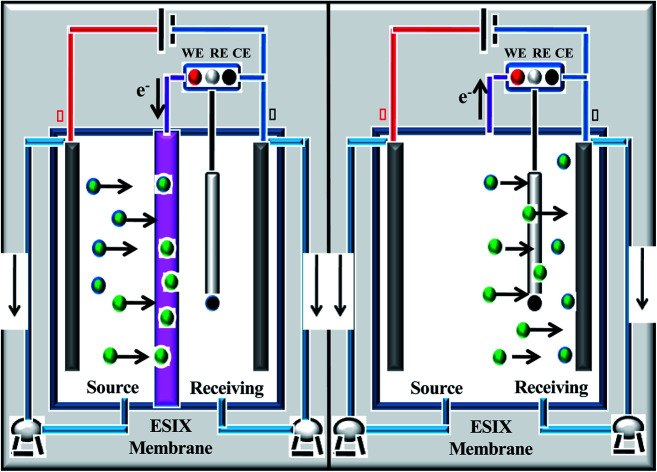

## 1. Introduction

Selective ion separation is an important process in the human body, which occurs during the removal of toxic ions that interfere physiological functions. It is also essential for water softening and desalination, wastewater purification and resource recovery in many industries.^1–3^ Traditionally, ion separation can be achieved through adsorption, oxidation/reduction, extraction, chemical precipitation and coagulation–flocculation. However, these methods either create significant secondary waste during the regeneration of the resin or cannot remove ions at low concentrations.^4–7^ Accordingly, the development of green and effective ion separation techniques is of significant interest from the perspective of resources and the environment.

Electrochemically switched ion exchange (ESIX) technology is an efficient and environmentally benign ion separation method. It not only has a reduced carbon footprint and secondary pollution, but also facilitates the operations in industry. Besides, the utilization of electricity as the driving force enables rapid ion separation even in dilute solutions.^8–10^ Therefore, ESIX has attracted wide attention and experienced rapid growth in the past few years. As film-based ion separation technology, the reversible uptake and release of the target ions in the ESIX process are achieved by electrochemically adjusting the redox state of the electroactive ion exchange materials. Considering that the efficiency of ion transportation is determined by the ion selectivity of the films, many efforts have been made to prepare films with desirable compositions and structures.^11–14^ To date, various electroactive materials have been developed and applied in the ESIX technique, including inorganic metal hexacyanoferrates such as CuHCF and NiHCF for Cs^+^, organic conducting polymers such as polypyrrole (PPy) for ClO^4−^ and polyaniline (PANI) for F^−^, and organic–inorganic hybrid materials such as PANI/a-ZrP film for Pb^2+^ and FeHCF/PPy electrodes for Na^+^ and Cl^−^.^15–18^ However, in the ESIX process, the selective ion separation and regeneration have to be performed separately.

To realize continuous ion separation, the ESIX technique has been combined with the ion membrane separation technique to develop an electric-driving ion-permselective membrane system.^19^ In the early development stage of this system, ions were selectively transferred from a dilute compartment to a concentrated compartment through an ion exchange membrane driven by a cell voltage applied on the sides of the system.^20,21^ Later, Kaya *et al.* employed a constant DC electric current to replace the electric voltage in the system, which resulted in significant improvements in the stability and efficiency.^22^ Considering that the abovementioned processes are batch operations and difficult to apply in industry, Weidlich *et al.* designed a semicontinuous operation cell, where two identical cation exchanger electrodes were divided by an anion exchange membrane and successfully removed Ca^2+^ and Mg^2+^ for water softening.^23^ However, it still has some defects, which require the electrodes to be reversed and the fluids in the compartments be exchanged periodically. Thus, to solve this problem, subsequently, Wallace *et al.* developed a series of continuous cell systems with an ion-permselective membrane, which has been widely used in ion separation research to date.^24–27^

Inspired by this, our group recently developed a novel electric field-accelerated ion-sieve membrane system based on the potential-oscillation technique.^28,29^ In this system, a constant voltage on a pair of auxiliary electrodes offers a stable external electric field to guide the migration of the target ions. Meanwhile, a pulse potential is applied to the membrane, which controls the uptake and release of ions by adjusting the redox state of the electroactive materials. Under the synergistic effect of these two structures, the ion selectivity and permeability are enhanced simultaneously, which has been successfully applied for the selective separation of target ions.^28,29^ Although, to date, ion separation systems are well-developed and have made encouraging progress, the integrated influence of various operating parameters such as the cell potential, pulse potential and placement of the electrodes on the separation performance is still not clear.

Herein, five common cell systems were selected and compared systematically to investigate the effects of the operating parameters on the ion transportation efficiency. The conducting polypyrrole (PPy) doped with polystyrene sulfonate (PSS^*n*−^) was applied as the electrically switchable cation exchange material. Moreover, stainless steel wire mesh (SSWM) was an ideal substrate to fabricate the PPy/PSS/SSWM membrane. Thus, the prepared membrane presented not only good conductivity and mechanical properties, but also sustainability due to the anti-corrosion property of PPy during the redox process.^30^ Furthermore, the permeability of potassium ions was used to evaluate the efficiency of these systems. The mechanism and the effects of the operation parameters including the cell voltage and the pulse potential on the membrane were intensively analyzed.

## 2. Experimental

### 2.1 Materials and instruments

Sodium polystyrenesulfonate (NaPSS) with an average molecular weight of 70 000 g mol^−1^ and pyrrole were purchased from Sigma-Aldrich. Pyrrole was purified through distillation before use. To remove the oxidized impurities, the distillate at 50–60 °C/66 kPa was collected for further polymerization. Other chemicals were purchased from National Medicine Group Chemical Reagent Co., Ltd., China and directly used without further purification. Aqueous solutions were prepared using ultrapure Millipore deionized water (18.2 MΩ cm). An SSWM (400 mesh) was applied as the conductive substrate.

Electrochemical experiments were carried out using a VMP3 potentiostat (Princeton Applied Research, USA) at room temperature. SEM and EDS photos of the PPy/PSS/SSWM membrane were taken using a scanning electron microscope (SEM, Hitachi SU8010, Japan) equipped with an energy-dispersive X-ray spectroscopy (EDS) detection system. The changes in the cation concentration in the aqueous solution were recorded *via* ion chromatography (DX-600, DIONEX, USA).

### 2.2 Preparation of PPy/PSS/SSWM membrane

SSWM (4 cm × 4 cm, 400 mesh) was pretreated sequentially with anhydrous ethanol for 1 h, 0.1 M sulfuric acid for 10 min, and deionized water to remove the residues and vacuum dried prior to electrodeposition. The electrodeposition reaction was performed in a three-electrode cell in conjunction with a VMP3 potentiostat, where an SSWM was used as the working electrode, a platinum plate was used as the counter electrode, and a saturated calomel electrode served as the reference electrode. The PPy/PSS membrane was electrodeposited on the surface of the pretreated SSWM *via* the potentiostatic method at a constant potential of 0.8 V for 1 h in a freshly prepared solution containing 0.2 mol L^−1^ pyrrole monomer and 0.1 mol L^−1^ NaPSS. Because the PPy became a cationic polymer after oxidization polymerization, large PSS^*n*−^ counter anions could be incorporated into the membrane to maintain electrical neutrality. Furthermore, it is known that the ionic permeability is related to the porosity, density, distance between polymer chains and electrostatic cross-linking.

### 2.3 Preparation of ion separation system

**Fig. 1:**
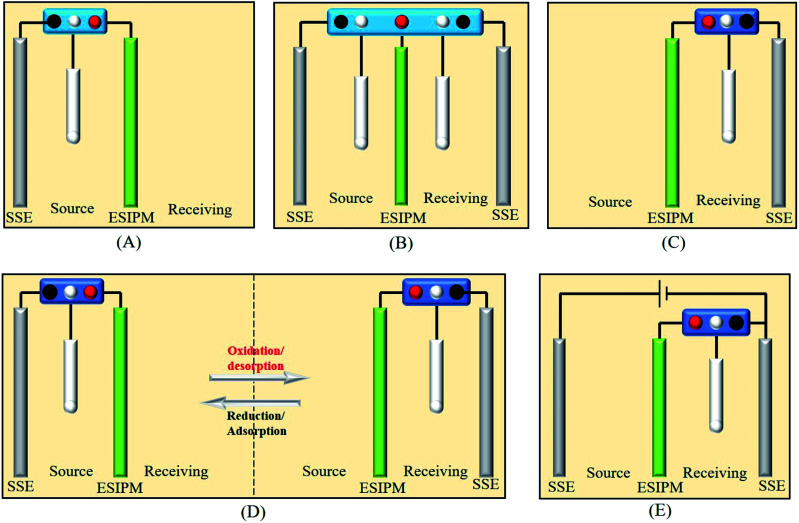
Five different ion separation systems.

To measure the efficiency of the ion separation systems, a set-up consisting of a VMP3 potentiostat, PPy/PSS/SSWM membrane, source compartment and receiving compartment was designed and set up, as shown in Fig. 1. Each compartment had an effective area of 2 cm × 2 cm. Then, 200 mL of 1 mM KNO_3_ and ultrapure water were added to the two compartments, respectively. The changes in the ion concentration in the receiving compartment for the different ion separation systems were detected by ion chromatography. The permeability of the PPy/PSS/SSWM membrane is defined by the following equation:*α*_perm_ = *c*′/*c*where *c*′ and *c* are the concentration of the ionic species in the receiving and feed solutions, respectively, in units of g mol^−1^.

### 2.4 Characterization

Morphological characterization was performed using a scanning electron microscope (SEM, JSM-6510LV, JEOL, Japan). The crystalline structure was determined using an X-ray diffraction instrument (XRD, SmartLab, Rigaku, Japan). Electrical conductivity was determined using a four-probe resistivity tester (SZT-2A, Suzhou Tongchuang Electronics Co., Ltd., China). Cyclic voltammetry (CV) was performed in a three-electrode system with a multi-potentiostat (VMP3, Princeton Applied Research, America) controlled with the EC-Lab software. Adsorption and desorption kinetics were also investigated in the three-electrode cell. In the ESIX process, the electrochemical capture of K^+^ was carried out when a reduction potential was applied to the working electrode, in which a composite electroactive film with a diameter of around 2 cm was used as the working electrode. The cation concentration in the aqueous solution was analyzed using an atomic absorption spectrometer (AAS, TAS-990, Beijing Purkinje General Instrument Co., Ltd., China). The adsorption capacity of the composite electroactive film (*Q*) was calculated using the following equation:
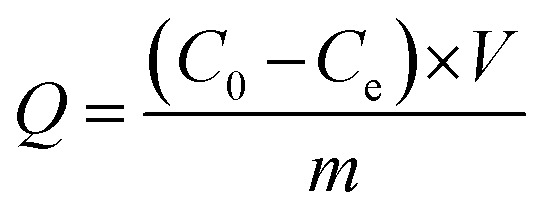
where *C*_0_ and *C*_e_ (mg L^−1^) are the initial and point-of-care K^+^ ion concentration during the testing, respectively; *V* (L) is the volume of the aqueous solution; and *m* (g) is the mass of composite electroactive film.

### 2.5 Current efficiency


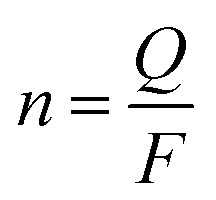
 
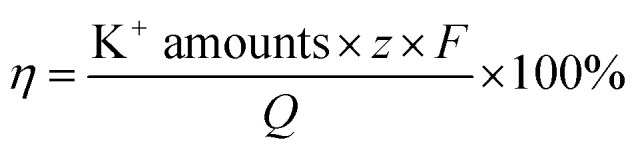
where *Q* is the quantity of electricity in a redox change unit (2 min) and *F* is the Faraday constant, 96 500C mol^−1^, and *z* is the valence state of K^+^.

## 3. Results and discussion

### 3.1 Characterization of PPy/PSS/SSWM membrane

**Fig. 2:**
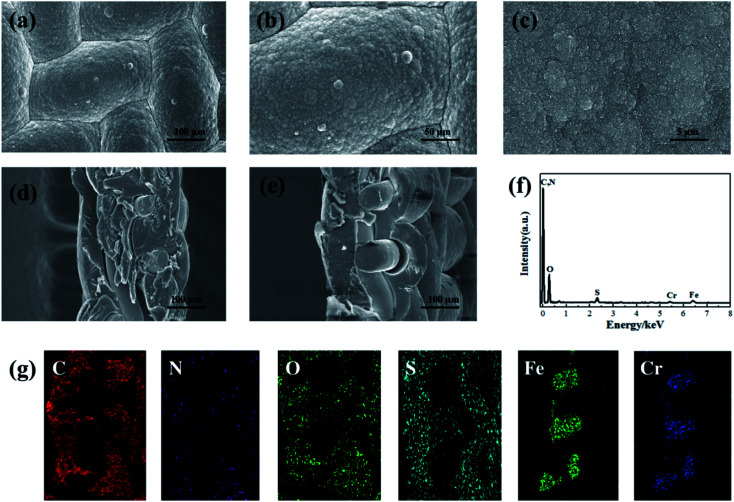
SEM images (a–e), EDS spectrum (f) and EDS mapping images (g) of the elements in the cross section of the PPY/PSS/SSWM membrane.

The morphology of the PPy/PSS/SSWM membrane was measured by SEM technology. As shown in Fig. 2a–c, the membrane has a smooth and dense surface. The images of the cross sections of the membrane (Fig. 2d and e) clearly show that the average thickness is around 130 um and the SSWM is completely wrapped in the PPy/PSS film. To identify its elemental composition, EDS investigations were further performed (Fig. 2f and g). In the EDS spectrum, the element C is assigned to the carbons in PPy and PSS, and the element N belongs to the pyrrole ring of PPy. The presence of Fe and Cr is from the SSWM. Moreover, the signals for the elements O and S prove the doping of PSS in PPy. The existence of PSS anions ensures the permselectivity of the PPy/PSS/SSWM membrane. The water absorption of the membrane material was examined, and it was found that its water absorption was 81 mg g^−1^. This good water absorption ability ensured that the metal ions in the solution could enter the membrane fluently. The results from the cyclic voltammetry investigation showed that the oxidation current and reduction current were symmetric, indicating that the redox process is reversible (Fig. S2†). The adsorption and desorption kinetics were also detected in the three-electrode cell (Fig. S2†). It was detected that the adsorption capacity of PPy/PSS/SSWM is 15.9 mg g^−1^, its desorption capacity is 13.0 mg g^−1^ and its recovery percentage is 81.8%.

### 3.2 Different types of electrical ion separation systems

**Fig. 3:**
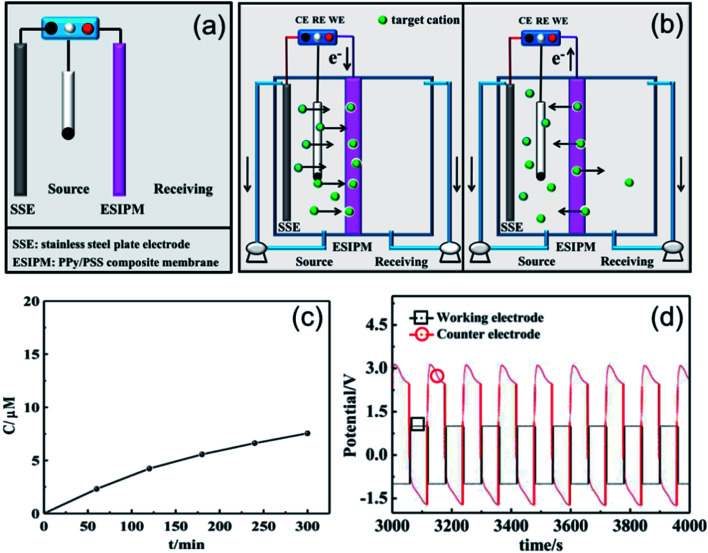
(a) and (b) Schematic illustration of the first ion separation system. (c) Concentration profile of K^+^ in the receiving solution. (d) Potential changes in the ion transport system.

The first ion transport system is a simple three-electrode control system, which was only assembled in the source room, as shown in Fig. 3a. A stainless steel electrode was used as the auxiliary electrode and the composite membrane PPy/PSS/SSWM served as the working electrode. 200 mL of 1 mM KNO_3_ and ultrapure water were added to the two compartments, respectively. A pulsed potential waveform (pulse width 60 s) from −1.0 V to +1.0 V was applied on the membrane working electrode.

The permeability of the membrane was used to determine the efficiency of the system and the changes in the ion concentration in the receiving room were detected by ion chromatography (Fig. 3c). It was found that the concentration of K^+^ increased with time and reached approximately 8 μM after running for 5 h, but the transfer rate gradually decreased. To further explore the ion transport mechanism in the system, the potential changes on the working electrode and auxiliary electrode (*U*) were measured using an electrochemical workstation. As shown in Fig. 2d, when a potential of −1.0 V was applied to the working electrode, a potential gradient of approximately +3.5 V was formed in the source room. Therefore, the cations under the electrical field driving force moved towards the membrane. When the ions arrived at the membrane, they were stored there temporarily due to the synergistic effects from the electrostatic interactions and propelling forces. Considering that the uptake/release of the target ions can be achieved by modulating the redox states of the membrane, an oxidized potential of +1.0 V was then applied to the working electrode. At this stage, a reversed potential difference of approximately 2.5 V was detected. Considering that there was no potential difference in the receiving room, the formed electrical gradient would repel most of the cations back to the source compartment and only a small portion of the target ions diffused into the receiving solution (Fig. 3b).

**Fig. 4:**
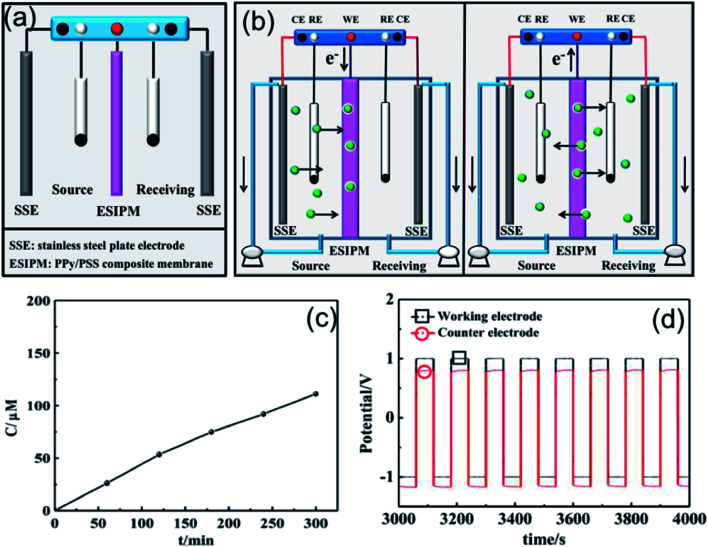
(a) and (b) Schematic illustration of the second ion separation system. (c) Concentration profile of K^+^ in the receiving solution. (d) Potential changes in the ion transport system.

When two auxiliary electrodes were both placed into the two compartments (Fig. 4a), an obvious difference in permeation flux, which is defined as the concentration of the ions passing through unit area in unit time, was observed. As shown in Fig. 4c, the concentration of K^+^ in the receiving room gradually increased and reached nearly 120 μM in the given time of 5 h, which was 15 times the absorption amount in the first system. Similarly, the potential changes were measured in an electrochemical workstation to investigate the ion transport mechanism (Fig. 4d). It can be seen that the potential gradient during the ion transportation process was only −0.25 V. Thus, the driving force derived from the potential field in the source room was not strong enough to transfer the cations to the composite membrane. When an oxidized potential of +1.0 V was applied to the membrane electrode, a potential difference of +0.25 V was detected. Considering that the attraction forces induced from the two auxiliary electrodes were equally applied to the cations in the membrane, the cations were finally distributed to the source room and receiving room evenly, which affected the overall efficiency (Fig. 4b).

**Fig. 5:**
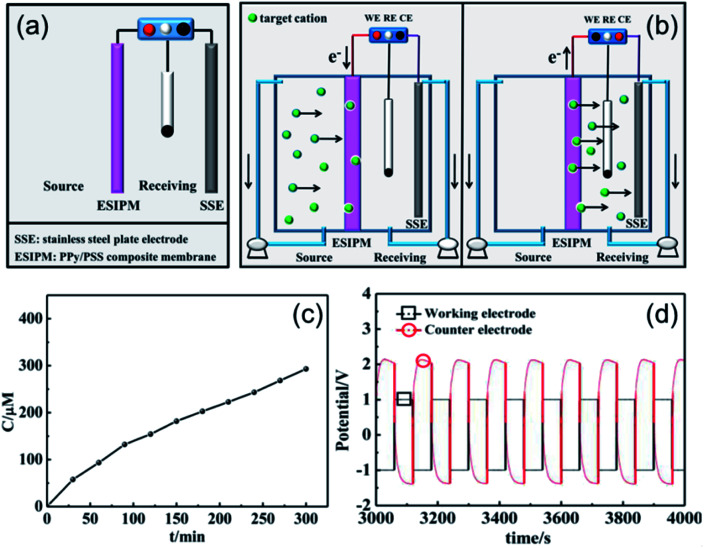
(a) and (b) Schematic illustration of the third ion separation system. (c) Concentration profile of K^+^ in the receiving solution. (d) Potential changes in the ion transport system.

When the three-electrode system was assembled in the receiving room only, the ion separation system was more effective compared with the abovementioned systems (Fig. 5a). As shown in Fig. 5c, the concentration of K^+^ increased with time. After running for 5 h, around 300 μM of target ions was collected in the receiving room, which was nearly three times the absorption amount in the second system. This phenomenon can be explained by the results from the potential changes on the working electrode and counter electrode (Fig. 5b and d). When a potential of −1.0 V was applied to the working electrode, the membrane was in the reduction state. Consequently, the electrostatic attractions could absorb and lock the diffused cations into the membrane. To release the ions to the receiving compartment, an oxidized potential of +1.0 V was then applied to the working electrode and a positive potential difference of approximately 2.5 V was found between the working electrode and the auxiliary electrode. Therefore, the cations under the electrical field driving force were repelled from the membrane and moved to the receiving solution.

**Fig. 6:**
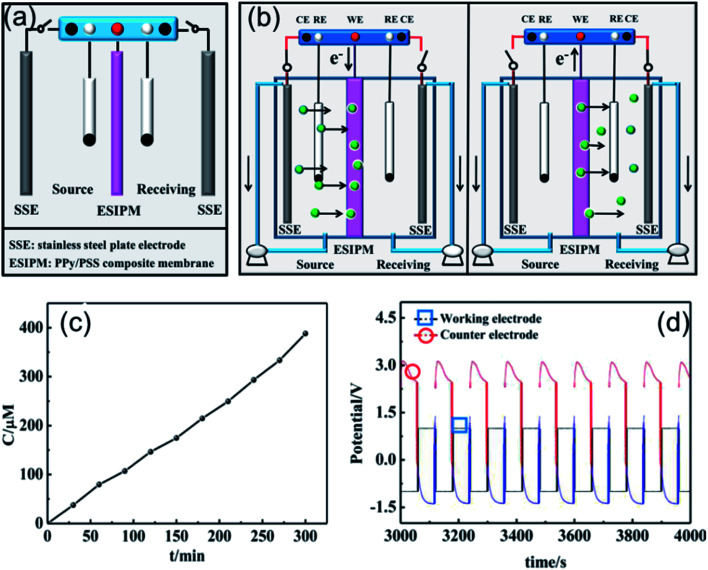
(a) and (b) Schematic illustration of the forth ion separation system. (c) Concentration profile of K^+^ in the receiving solution. (d) Potential changes in the ion transport system.

Based on the above analysis, the ideal condition would be the combination of the first and third ion separation systems. Therefore, an ion transport system with two switches was designed, as shown in Fig. 6a. To determine the efficiency of the system, the changes in the concentration in the receiving room were detected by ion chromatography. As shown in Fig. 6c, the concentration of K^+^ increased with time and reached approximately 400 μM in the given time, which is consistent with our expectation. Moreover, the ion transport mechanism was a combination of that in the abovementioned systems, where the +3.5 V potential difference formed in the source room could drive the cations to move towards the membrane and the +2.0 V potential gradient would attract the cations to the receiving solution (Fig. 6b and d). Due to its high efficiency, this system has been widely used in ion separation experiments.

There are three steps in the process of ion permeation through the membrane. Firstly, the ions migrate to the membrane surface on the side of the feed solution, then diffuse in the membrane, and finally released on the other side of the membrane. In the first system, the pulse voltage is only applied to one side of the source liquid. During adsorption, the potential is used as the driving force to accelerate the migration of ions to the membrane. During desorption, the reversed potential forces the ions to return to the source chamber, and thus the adsorption efficiency is low. To improve the desorption process, the potential is also added to the receiving chamber in the second system, which makes the ions diffuse to both chambers equally in the desorption process, thus partially enhancing the efficiency. The third system only applies a pulse potential to the receiving solution, and thus the adsorption process is inefficient. In the desorption process, the pulse potential is beneficial for the migration of ions to the receiving solution side with improved efficiency. The disadvantage is that the potential does not provide a favorable driving force on the side of the source solution in the adsorption process. To further improve the efficiency, we designed a forth system, which periodically changes the position of the potential addition, and thus the potential is added to the source chamber in the adsorption stage to facilitate ion migration. Besides, in the desorption stage, the potential is reversed to enhance the releasing efficiency to achieve a better adsorption effect.

**Fig. 7:**
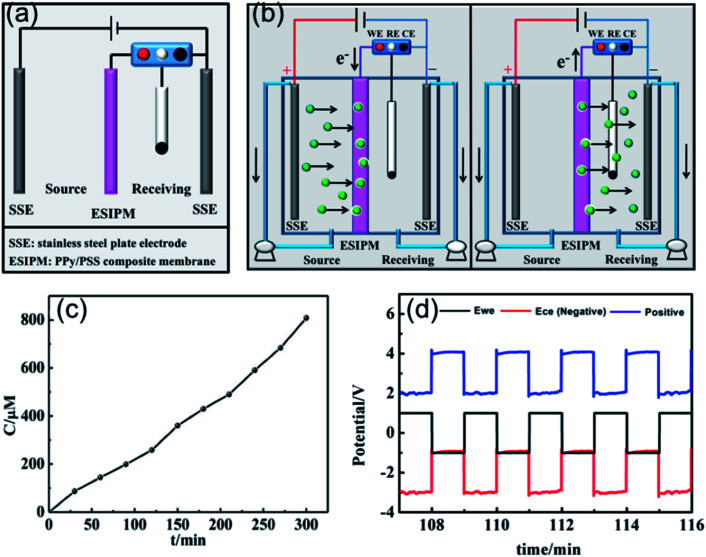
(a) and (b) Schematic illustration of the fifth ion separation system. (c) Concentration profile of K^+^ in the receiving solution. (d) Potential changes in the ion transport system.

Recently, inspired by the abovementioned ion exchange systems, an *in situ* electric field-accelerated ion sieve system was developed by our group. The optimized ion separation system consisted of a two-electrode system and one three-electrode system (Fig. 7a). A pulse potential (pulse width 60 s) provided by the three-electrode system was applied on the membrane working electrode. Meanwhile, a constant +5 V cell voltage provided by the two-electrode system could create a constant external electric field driving force. Considering that the uptake/release of the target ions can be achieved by modulating the redox states of the membrane, a pulse potential in the range of −1.0 V to +1.0 V was applied to the working electrode. As shown in Fig. 7c, the concentration of K^+^ in the receiving solution was approximately 800 μM after 5 h, which was almost 100 times that in system 1. Compared to the systems above, the significant difference is attributed to the use of the external electrode. It can be seen that when the potential of −1.0 V was applied on the working electrode, the potential difference of +5.0 V was detected in the source cell, which made the target ions directionally migrate to the membrane. However, when the working electrode potential was tuned to +1.0 V, the potential gradients in the source room and receiving room were equal potential volumes (Fig. 7b and d), respectively. Under this synergistic function, the *in situ* ion transport not only lead to an effective permeable flux of the target ions, but also ensured a stable and high ion transport rate.

### 3.3 Comparison of the electrical ion separation systems

**Fig. 8:**
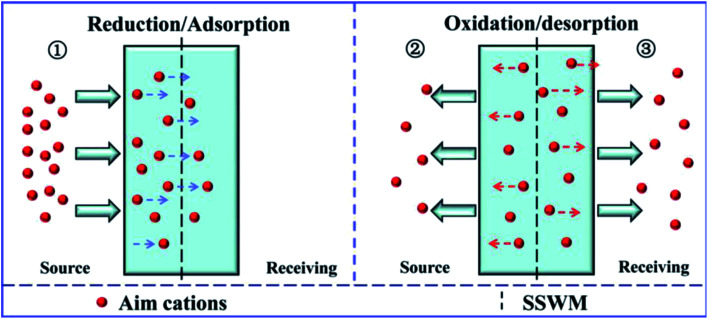
Schematic graph of the cation transport process through the PPy/PSS membrane.

According to the analysis above, it can be found that there are three processes for the ion transportation through the electrically switchable cation exchange membrane (Fig. 8). Initially, the cations in the feed solution are driven into the membrane, which is mainly affected by the reduction state of the membrane and the positive potential difference in the source chamber. In the second stage, some of the cations in the membrane are reversely repelled back into the source solution, which is mainly affected by the oxidation state of the membrane and the reverse potential difference in the source chamber. Meanwhile, the target cations left in the membrane are released into the receiving solution, which is determined by the oxidation state of the membrane and the driving force of the positive potential difference in receiving room. As the result of all these mechanisms, the target ions are successfully delivered into the desired receiving chamber. Besides, it is clear that the potential differences in the compartments are key factors that influence the system efficiency. As the positive potential difference increases, the ion transfer capacity increases (Table 1). The energy consumption and the ion transfer capacity were also calculated to evaluate the system efficiency. It can be seen that although the energy consumption in the fifth system is not the lowest, combined with the ion recovery efficiency and rate, it is still the most advantageous. Therefore, the electric field-accelerated ion sieve is the most optimized system for ion transportation.

**Table 1:** Comparison of the cations fluxes and transport driving forces in the five electrochemically switched ion transport systems

		1	2	3	4	5
Source room	Potential (oxidized state, V)	2.5	0.2	0	0	1
	Current (oxidized state, mA)	3	1.5	0	0	2.5
	Time (oxidized state, h)	2.5	2.5	0	0	2.5
	Potential (reduction state, V)	3.5	0.2	0	3.5	5
	Current (reduction state, mA)	3	1.5	0	6	0.5
	Time (reduction state, h)	2.5	2.5	0	1.25	2.5
Receiving room	Potential (oxidized state, V)	0	0.2	2.5	2.5	4
	Current (oxidized state, mA)	0	1.5	1	1	2.5
	Time (oxidized state, h)	0	2.5	2.5	1.25	2.5
	Potential (reduction state, V)	0	0.2	3	0	0
	Current (reduction state, mA)	0	1.5	1	0	0.5
	Time (reduction state, h)	0	2.5	2.5	0	2.5
Energy consumption (J)		162	10.8	49.5	105.8	135
Ion transfer capacity (μmol)		1.51	22.22	58.61	77.61	161.57
Energy consumption per μmol (J μmol^−1^)		107.3	0.5	0.8	1.4	0.8
Ion transfer rate (μmol s^−1^)		8.39 × 10^−5^	1.23 × 10^−3^	3.26 × 10^−3^	4.31 × 10^−3^	8.98 × 10^−3^

### 3.4 Equivalent circuit analysis of the electric field-accelerated ion sieve system

**Fig. 9:**
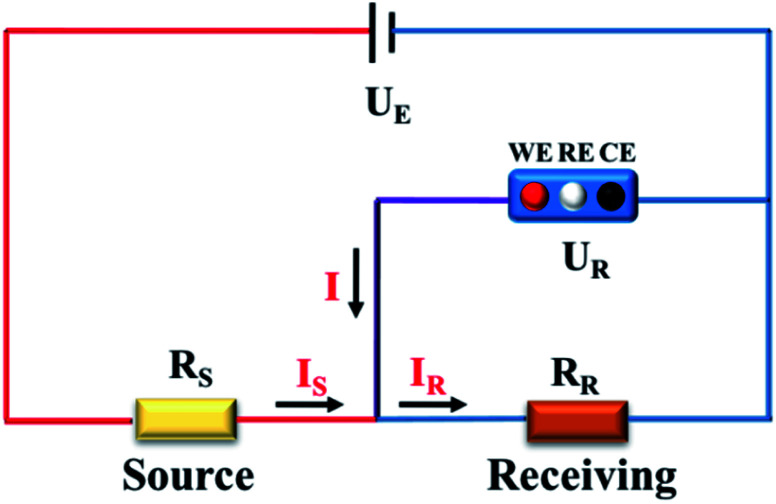
Equivalent circuit of the *in situ* electric field-accelerated ion transport system.

To get a better understanding of the ion transport mechanism in the fifth system, an equivalent circuit is illustrated in Fig. 9. It the resistivity of the membrane was measured to be 5.15 × 10^−1^ Ω cm, which indicates that the film has good electrical conductivity. Therefore, the membrane resistance was neglected in the calculations. *U*_E_ is the cell voltage applied to the two-electrode system and *U*_R_ is the potential difference in the receiving room. According to the analysis above, it is known that both the cell voltage and the pulse potential are responsible for the ion migration. When the membrane is in its reduction state, the source potential, namely *U*_E_–*U*_R_, contributes to the ion permeable flux. When the membrane is in its oxidation state, the receiving potential, *U*_R_, will be responsible for the release of the target ions. If the resistances in the two cells are defined as *R*_R_ and *R*_S_, respectively, the currents can be calculated using the following equations:

When the membrane is in the reduction state:1
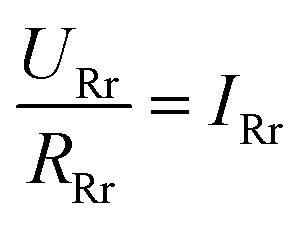
2
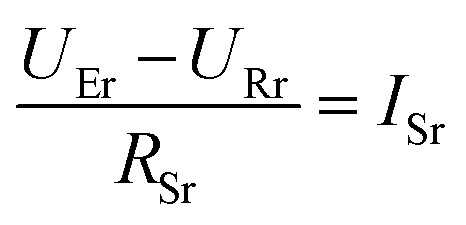

3 *I*_r_ + *I*_Sr_ = *I*_Rr_

4
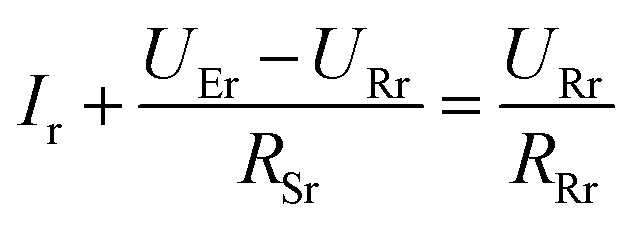


When the membrane is in the oxidation state:5
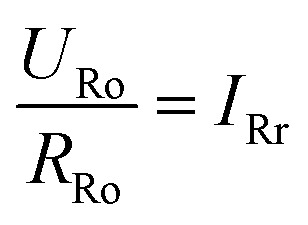
6
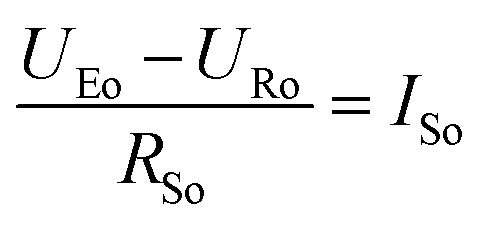

7 *I*_o_ + *I*_So_ = *I*_Ro_

8
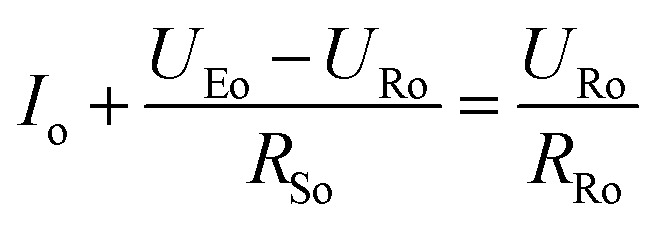


Considering that the ion concentrations in the two rooms are almost constant in each redox modulating unit, it is assumed that *R*_R_ and *R*_S_ are same. Therefore,

9 *R*_Rr_ = *R*_Ro_ = *R*_R_

10 *R*_Sr_ = *R*_So_ = *R*_S_

Thus, *R*_R_ and *R*_S_ can be calculated as follows:11
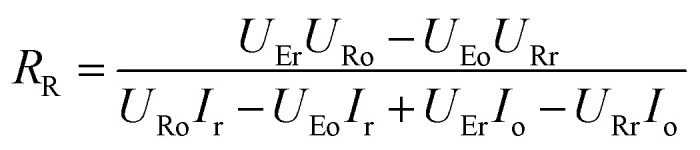
12
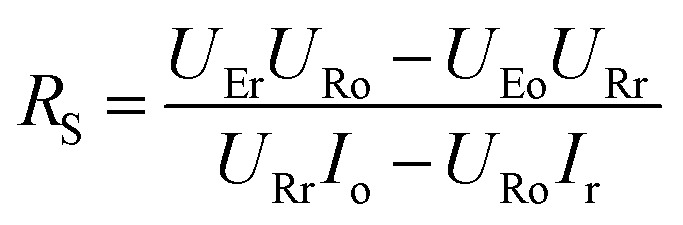


It was detected that *U*_Rr_ is 0 and *U*_Ro_ is 4 V. Conversely, *U*_Er_ is 5 V and *U*_Eo_ is 5 V (Fig. 6d). Therefore, the currents in the circuits at different redox states are defined by the following equations:

13 *I*_Rr_ = 0

14 *I*_Sr_ = −*I*_r_

15
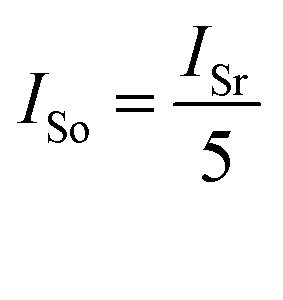

16 *I*_Ro_ = *I*_o_ + *I*_So_

According to these calculations, it is speculated that there was almost no current in the receiving solution circuit when the membrane is reduced.

### 3.5 Current efficiency

**Fig. 10:**
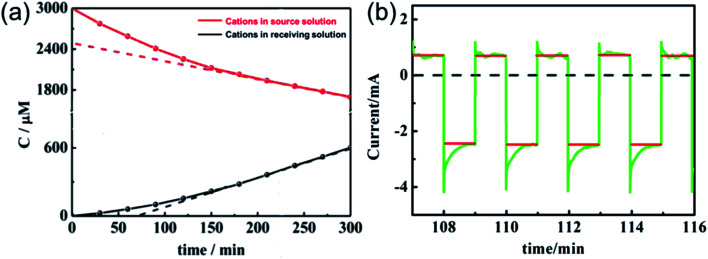
Current changes (a) in the source and receiving solution and (b) on the membrane of the *in situ* electric field-accelerated ion transport system. The green lines represent the pulse current and the red lines are the average value of the current.

The current efficiency of the *in situ* electric field-accelerated ion transport system was determined based on a K^+^ transport experiment, where the pulse potential applied to the membrane was −1 V to 1 V, the pulse width was 60 s, and cell voltage for 5 h. 200 mL of 1 mM K^+^ solution was pumped into the source chamber and 200 mL of deionized water was pumped into the receiving chamber. As shown in Fig. 10a, the ion concentration in the source room decreased with time and the flux rates gradually slowed down. In comparison, the ion concentration rapidly increased in the receiving room. With an increase in K^+^ in the receiving solution, the growth tendency became slow and finally remained constant after 2 h. Moreover, given that there is a difference between the ion quantities moving in and out of the membrane, it can be seen that the ions gradually accumulated in the membrane and reached a saturated state after 2 h, where the ion uptake and release rates were almost same.

It is known that the current through the membrane changes in accordance with the redox state changes in electroactive membrane. Therefore, the current profile was recorded on the membrane of the ion separation system (Fig. 10). When a potential in the range of −1 V to +1 V was applied to the membrane, +0.5 mA reduction current and −2.5 mA oxidation current were detected. Considering that the reduction current is 20% of the oxidation current, this result is consistent with the previous calculations. Moreover, it can be calculated from the area integral that the quantities of electricity within a redox modulating unit is 0.04144C and 0.1621C, respectively. Accordingly, the ion removal amount was calculated to be 1.68 × 10^−6^ mol and the current efficiency, *η*, to be 81%. Compared with the traditional electrochemically controlled salt removing system, capacitive deionization, the designed system displays not only a high removal speed, but also a surprisingly high efficiency. In addition, given that *Q*_r_ is much less than *Q*_o_, it is considered that the electricity consumption during the ion absorption is the main factor that influences the current efficiency (Fig. S1†).

## 4. Conclusion

A novel and effective electric field-accelerated ion sieve system was designed for ion separation. In this system, the membrane material was fabricated by coating PPy/PPS on stainless steel wire mesh. Meanwhile, a constant cell voltage and a pulse potential on the membrane worked synergistically to improve the ion transportation rate and permeability efficiency. In comparison with the other four common systems, it was revealed that the potential-enhanced ion transport system has the highest ion flux in the receiving compartment, which was almost 100 times that in only the left-chamber assembled system. Furthermore, it was proven that the positive potential gradients in the source room and receiving room were the main factors affecting the ion transfer efficiency. For a quantity analysis of the system efficiency, an equivalent circuit was illustrated, and up to 81% current efficiency was calculated. Obviously, the proposed ion separation system has significant potential for the continuous separation of the target ions in dilute solution by selecting appropriate materials, which can be applied in industrial wastewater treatment.

## Conflicts of interest

There are no conflicts to declare.

## Supplementary Material

RA-011-D1RA00924A-s001
