# Supplementary material for: Investigation of a novel high-efficiency ion-permselective membrane module based on the electrochemically switched ion exchange scheme
Source: RSC Adv. 2021 Jun 16;11(35):21248–58. doi: 10.1039/d1ra00924a (PMC9034045; doi:10.1039/d1ra00924a)
Supplement: RA-011-D1RA00924A-s001 [file RA-011-D1RA00924A-s001.pdf]

Electronic Supplementary Material (ESI) for RSC Advances.
This journal is © The Royal Society of Chemistry 2021

# Investigate of a novel high-efficiency ion permselectivity membrane module based on ESIX scheme

Di Zhang [a], Pengle Zhang [b], Du Xiao [b], Xiaogang Hao [b,*]

[a] Department of Chemistry, Shanxi Medical University, 56 Xinjian South Road, Taiyuan, 030001, China

[b] Department of Chemical Engineering, Taiyuan University of Technology, Taiyuan, 030024, China

[*] Corresponding author.

E-mail: xghao@tyut.edu.cn (X. Hao)

Tel.: + 86 351-6018193; Fax: + 86 351-6018554;

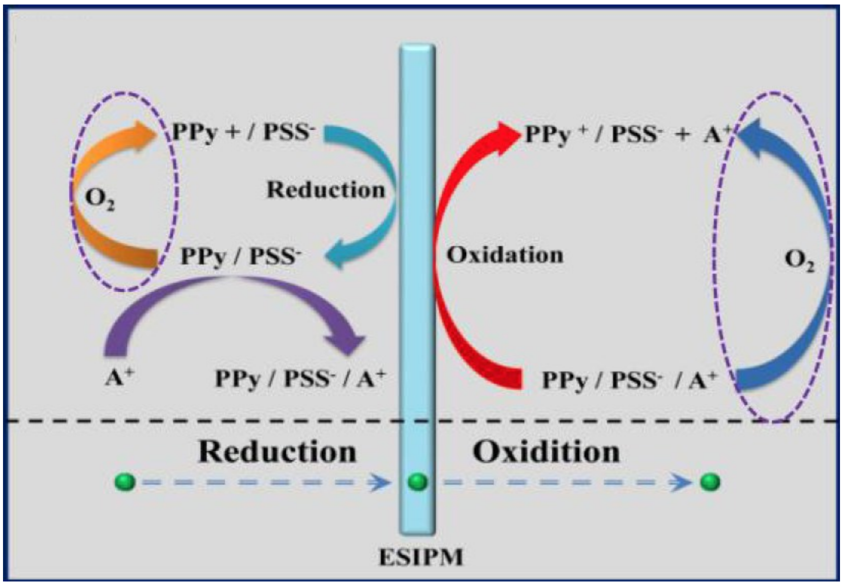


**Figure S1.** Effect of the dissolved oxygen in the solutions on the cations transport through the PPy/PSS membrane.

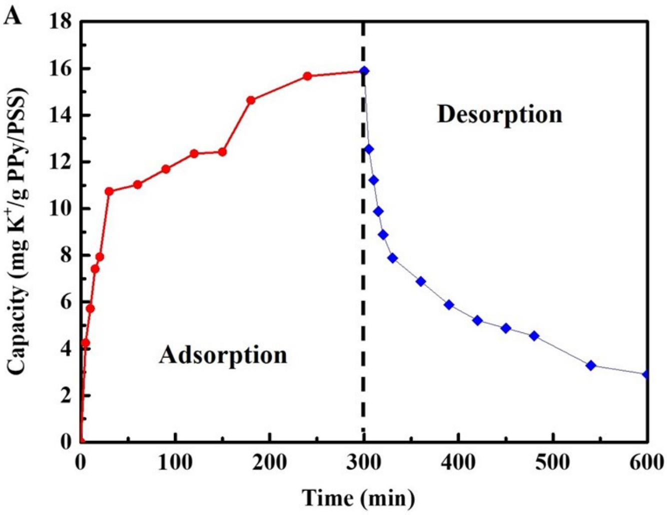

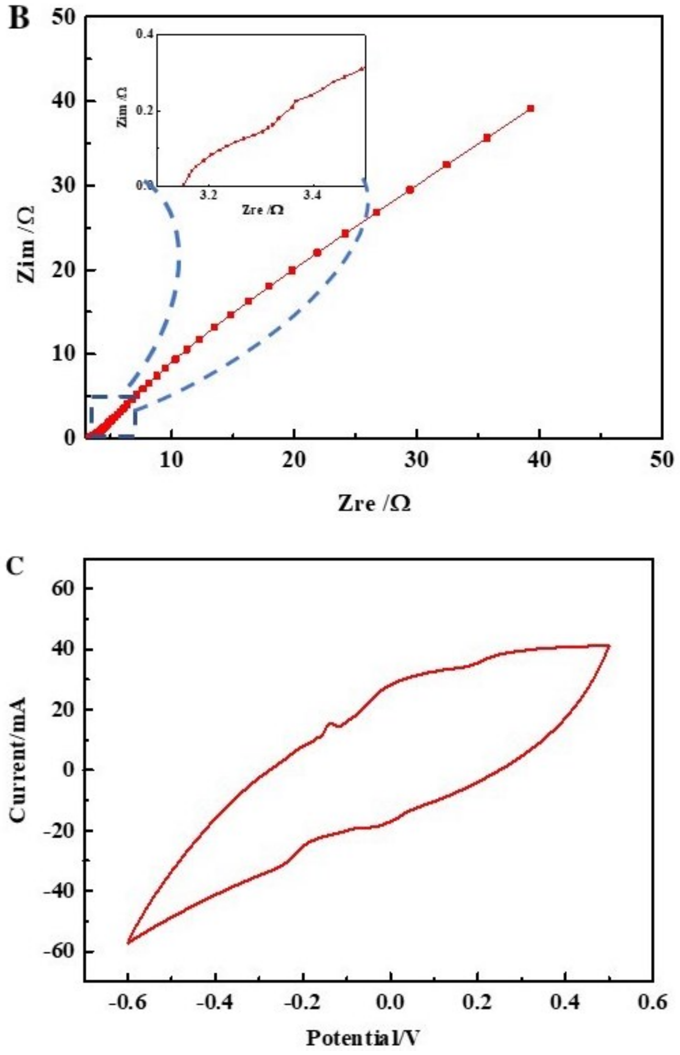


**Figure S2. (A)**The adsorption and desorption curves of $K^+$ ions on the PPy/PSS membrane; (B)The impedance of the membrane; (C) CV curves of the PPy/PSS membrane as electrode.
